# Comparison of Psycho-Social Factors Associated With Suicidal Ideation and Suicide Attempts Among People Living With HIV in Central West China

**DOI:** 10.3389/fpubh.2022.832624

**Published:** 2022-03-25

**Authors:** Xuelian Wang, Chuanyuan Yan, Yongsheng Tong, Juan Gao, Wei Zhou, Zhichao Lan, Jianlan Wu, Hongbing Li, Yi Yin, Yuehua Wang, Nancy H. Liu, Feng Deng

**Affiliations:** ^1^Peking University HuiLongGuan Clinical Medical School, Beijing, China; ^2^Beijing Suicide Research and Prevention Center, Beijing HuiLongGuan Hospital, Beijing, China; ^3^World Health Organization Collaborating Center for Research and Training in Suicide Prevention, Beijing, China; ^4^Baoji Center for Disease Control and Prevention, Baoji, China; ^5^Center of Disease Control and Prevention of Meixian County, Baoji, China; ^6^Department of Psychology, University of California, Berkeley, Berkeley, CA, United States

**Keywords:** HIV, suicide attempt, suicide ideation, mental health, China

## Abstract

**Background:**

Previous studies have described the correlation of suicidal ideation (SI) or suicide attempts (SA) in people living with HIV (PLWH), whereas few studies compare the correlation between SI and SA in PLWH. Understanding specific risk factors for SI and SA among PLWH will help with developing tailored and effective suicide prevention strategies among this high-risk group.

**Methods:**

A cross-sectional study was conducted from December 2020 to April 2021 in Baoji municipality, Shaanxi Province. The PLWH registered with the Baoji Municipal Center for Disease Control and Prevention (CDC) were recruited and interviewed. Questionnaires and interviews for this study consisted of socio-demographic variables, mental health history, and psychosocial characteristics. The HIV-related clinical features were obtained from CDC medical records. The PLWH included were divided into three groups, i.e., those with a history of suicide attempts (SA group), those with suicidal ideation only (SI group), and those without any suicidal behavior (NSB group). Multinomial logistic regression was used for three-way comparisons among these three groups of PLWH.

**Results:**

In total, 995 PLWH were interviewed. The prevalence of probable depression, probable anxiety, SI, and SA in PLWH after being diagnosed as HIV+ was 18.6%, 13.5%, 26.7%, and 3.2%, respectively. Compared with the NSB group, the SI or SA groups were more likely to report probable depression [adjusted odds ratio (AOR) = 2.43, 4.44, respectively], probable anxiety (AOR = 2.80, 5.62, respectively), and high HIV-related stigma (AOR = 2.05, 2.65, respectively). The SI group was more likely to experience high HIV-related stress (AOR = 1.91) and lower quality of life (AOR = 0.56) than the NSB group. Social support and HIV-related clinical features were not associated with SI or SA in this sample. The SA group did not differ from the SI group on any of the psychosocial or HIV-related clinical features.

**Conclusions:**

Mental health problems are serious in community residents identified with having an HIV infection in a Central West China municipality. It is important to deliver low-cost and effective psychological services tailored for PLWH that are focused on reducing mental health problems. Future studies should utilize sensitive screening measures and further clarify factors potentially associated with the transition from SI to SA in PLWH.

## Introduction

The Human Immunodeficiency Virus (HIV) infection is a serious public health problem. At the end of September 2018, China had over 840,000 people living with HIV (PLWH) (1). With the increased availability of antiretroviral therapy (ART), the life expectancy in PLWH is extended (2). Yet, HIV-related stigma and long-term stress can lead to more mental health problems, including depression, anxiety, and suicidality, in PLWH compared with the general population (3, 4).

Most previous studies on the mental health of PLWH have limited their focus to depression and anxiety. Although suicidality is a serious problem among PLWH, few studies have focused on suicide death, suicide attempt (SA), and suicidal ideation (SI) in this population (5). Worldwide, the incidence of suicide death and lifetime prevalence of SA and SI were substantially higher in PLWH than in the general population (6). In China, among PLWH, suicide is one of the leading causes of non-AIDS death (7). Suicide mortality is 20.9 times greater in PLWH than in the general population (8) and the prevalence of SA in the past year ranged from 3.8 to 8% (5). One study found that in Changsha municipality in Central China, 25% of PLWH had SI following their HIV diagnosis (9).

Several studies have reported PLWH-specific correlation to SI. HIV-related clinical features associated with SI after individuals were identified as HIV-positive included CD4 lymphocyte counts and transmission route (9, 10), symptoms of mental health conditions (9–11), HIV-related stress, lack of social support, and other social factors (9, 12, 13). More limited literature has examined the PLWH-specific correlation between SA and suicide death. Depression and less social support were associated with SAs that occurred in the 6 months after HIV diagnosis (14). Using a registered linkage study, Jia et al. (15) identified high comorbidity of psychiatric illness among individuals with AIDS or HIV infection, and this comorbidity substantially increased the risk of suicide death in PLWH compared with those without any mental health comorbidity.

Although in most cases SA occurs with preceding SI, only a few individuals with SI ultimately carry out fatal or non-fatal self-harm acts (16). Clarifying the correlation between SI and SA among PLWH would contribute to our understanding of why some only have SI while others engage in SA. Previous studies have separately focused on the correlation of SI or SA in PLWH, with no noteworthy differences between correlation of SI vs. SA. Clarifying these factors will help us develop and refine targeted suicide prevention efforts geared at PLWH.

In our present study, we recruited registered PLWH in Baoji, a Central West municipality in China. Our study aimed to compare the characteristics including mental health conditions, HIV-related clinical features, HIV-related stigma and stress, social support, and quality of life among three groups of PLWH, i.e., those with SA, those with SI only (did not carry out self-harm acts), and those with neither SI nor SA or the no suicidal behavior (NSB) group, after they being identified as HIV-positive.

## Methods

### Participants

The study was conducted in Baoji municipality, a middle-income area in the west of Shaanxi Province. The per capita gross domestic product (GDP) of this central-western province ranks in the middle of the 31 provinces in mainland China. Participants were residents diagnosed with HIV/AIDS infection. The recruitment was conducted by the Baoji Municipal Center for Disease Control and Prevention (CDC) from 31 December 2020 to 1 April 2021. The inclusion criteria were as follows: (a) registered in Baoji CDC system as having an HIV/AIDS infection, (b) residing in Baoji municipality, and (c) aged 18 years or older. We excluded individuals (a) with a severe mental disorder, intellectual disability, or other communication problems, (b) who were unable to be contacted, and (c) who refused to participate in the study. At the end of 2020, a total of 1,266 people were invited to participate. After excluding according to the exclusion criteria, a final total of 995 individuals were included in the study.

### Procedure

The study was approved by the institutional review board of the Beijing HuiLongGuan Hospital, and written informed consent was obtained from all participants. Data collection consisted of self-reported questionnaires and HIV infection records from the Baoji CDC system. According to the CDC registration mechanism of PLWH in China, all confirmed cases of HIV/AIDS are registered with the local CDC. For this study, registered PLWH living in Baoji Municipal was contacted by the Baoji CDC staff by telephone. The recruited PLWH were interviewed face-to-face. Trained Baoji CDC staff administered self-report questionnaires. Interviews were conducted in a standardized fashion. At the end of each interview, interviewers would check responses to ensure data quality (e.g., eliminating errors, unnecessary missing data, etc.).

### Measures

#### Suicidality: Ideation and Attempts

Suicidal ideation was defined as seriously thinking of killing oneself, and suicidal attempts were defined as acts of self-harm with the intent to kill oneself (16). In our study, two questions were used to assess participants' suicidal ideation and attempts after being diagnosed with HIV/AIDS infection. Participants responded on a 3-point Likert-type rating scale (0=no, 1=rarely, 2=sometimes, and 3=always) to the following questions: (a) since you were diagnosed with HIV/AIDS infection, how often have you ever thought of killing yourself? and (b) how many times did you make a suicide attempt after being diagnosed with HIV/AIDS infection?

If the participant reported having made an SA at least once, he/she was classified as with a suicidal attempt (SA). Respondents who endorsed SIs but denied SAs were classified as having suicidal ideation only (SI). Participants who denied SA and SI were classified as without SI or SA (NSB).

#### Socio-Demographic Characteristics

Socio-demographic characteristics that were collected in our study included the following: age (divided into three age groups: 18–29, 29–39, ≥40 years), sex (male or female), marital status (single, married, or widowed /divorced), residence (rural or urban), ethnicity (Han Chinese or others), religious beliefs (no religious belief, Buddhism, other religious beliefs), educational level (middle/technical secondary school or lower, high school/vocational high school, college/university or above), work status (employed or self-employed, unemployed, others), annual income per capita [renminbi (RMB) <40,000, ≥40,000], and living situation (alone or with others).

#### HIV Infection Record

The HIV infection records of participants were obtained from the Baoji Municipal CDC system. This included HIV transmission [same-sex sexual history, other (e.g., heterosexual sexual behavior, injection drug use, others)], same-sex sexual history (yes or no), venereal disease history (i.e., one that had sexually transmitted disease), AIDS diagnosis (i.e., diagnosed with AIDS, or HIV infection only), and CD4 counts (<200 cells/mm^3^, 200–500 cells/mm^3^, >500 cells/mm^3^).

#### Psychosocial Factors

Several psychosocial factors including depression, anxiety, HIV-related stress and stigma, social support, and quality of life were collected for this study. Self-reported measures using the Chinese versions of the Patient Health Questionnaire (PHQ-9) (17) and the Generalized Anxiety Disorder Scale (GAD-7) (18) were used to measure depression and anxiety, respectively. Participants reported how many times they had been bothered by the depression or anxiety symptoms in the last 2 weeks using a 4-point scale. Scores ≥10 on either the PHQ-9 or GAD-7 suggested the presence of depressive symptoms and/or anxiety symptoms, respectively. The Cronbach's α coefficients of PHQ-9 and GAD-7 in the present study were 0.92 and 0.95, respectively.

The HIV-related stress was defined as the extent to which one has felt stress specific to the HIV/AIDS infection and was measured using the Chinese version of the HIV/AIDS Stress Scale (CSS-HIV) (19). This measure has three dimensions: 1) emotional stress, 2) instrumental stress, and 3) social stress. Each item was rated on a 5-point Likert-type scale ranging from 0 (not at all) to 4 (extremely). Higher scores indicated greater stress. Scores within 0–13 were defined as low, whereas those of 14 or above were defined to have high HIV-related stress. The Cronbach's α coefficient for this measure of HIV-related stress in the present study was 0.92.

The HIV-related stigma was defined as a perceived stigma and was measured using the Chinese version of Berger HIV Stigma Scale (BHSS) (20, 21) which consisted of 15 items. This measure assesses four dimensions: 1) rejection of HIV patients, 2) disclosure concerns, 3) negative self-image, and 4) consequences of disclosure. Each item is marked as one with which respondents disagreed (scored 0) or agreed (scored 1). Higher scores indicated higher HIV/AIDS-related stigma. Scores within 0–11 were defined as low and those with 12 or above were defined as high HIV-related stigma. The Cronbach's α coefficient for this measure in the present study was 0.80.

The Chinese social support rating scale (SSRS) yields three dimensions of social support: 1) subjective support, 2) objective support, and 3) support-seeking behavior with 10 items (22). Higher scores indicate higher social support. Scores of 0–25 were defined as low social support and scores of 26 or above were defined as high social support. The Cronbach's α coefficient for this measure in the present study was 0.67.

The quality of life was measured by using the Chinese version of the Quality-of-Life Scale (6-items) consisting of six dimensions: 1) physical, 2) psychological, 3) financial status, 4) work, 5) relations with family, and 6) relations with others (23). Responses were measured using a Likert-type scale which ranged from 1 (extremely bad) to 5 (extremely good). Higher total scores indicated better quality of life. Scores of 0–15 indicated the low quality of life, while scores of 16 or above indicated the high quality of life. The Cronbach's α coefficient for this measure was 0.89 in our study.

#### Statistical Analysis

Participants were divided into three groups based on their responses, i.e., those with the suicidal attempt (SA) group, the suicidal ideation only (SI) group, and the no suicidal behavior (NSB) group. Descriptive statistics [e.g., median, interquartile range (IQR), proportion, and prevalence] of sociodemographic characteristics, HIV infection records, and psychosocial factors were analyzed for the total sample and compared among the three groups. Differences in sociodemographic characteristics, HIV infection records, and psychosocial factors among the three groups were examined using Chi-square or the Kruskal-Wallis tests. To reduce type I errors, a Tukey-type method for nominal variables and a non-parametric method for ranked variables were used to conduct multiple comparisons if the overall test reached statistical significance (24). A multivariable multinomial logistic regression model was used for three-way comparisons among three groups of PLWH. In multinomial logistic regression, we ran two sets of models. The first model assessed the risk for attempted suicide and SI associated with psychosocial factors (i.e., depression, anxiety, HIV-related stress, HIV-related stigma, social support, and life quality) after adjustment for sociodemographic variables. The second model additionally assessed the risk for SI and attempts associated with HIV-related clinical factors (i.e., HIV-transmission, AIDS diagnosis, venereal history, and CD4 counts) based on the first model. We performed all the statistical analyses using SPSS, version 25.0 for Windows.

## Results

### Participant Characteristics

**Figure 1:**
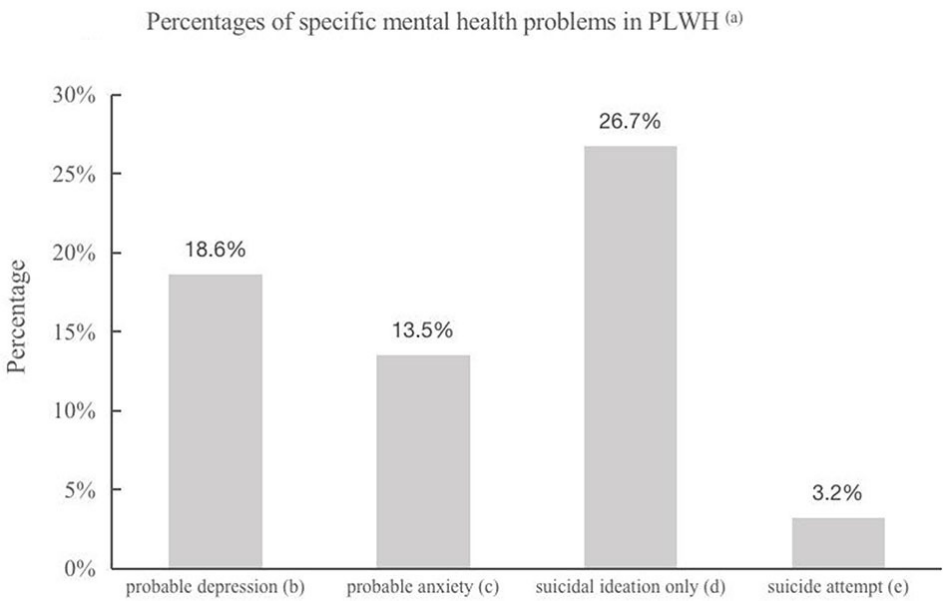
(a) PLWH: People Living With HIV. (b) Probable depression was defined as having a score ≥10 on Patient Health Questionnaire (PHQ-9). (c) Probable anxiety was defined as having a score ≥10 on the Generalized Anxiety Disorder Scale (GAD-7). (d) Participants who reported suicidal ideation without suicide attempt after HIV/AIDS infection. (e) Participants who reported occurrence of suicide attempt(s) after HIV/AIDS infection.

As noted above, a total of 1,266 unduplicated surviving PLWH registered in the Baoji Municipal CDC system. In total, 271 registered PLWH did not complete the interview for different reasons (3 with an intellectual disorder, 8 with age-related communication problems, 9 with severe mental disorders, and 251 could not be contacted). The final sample of 995 registered PLWH was included in the present study. Among them, 32 (3.2%) reported SAs, which occurred after they were identified as HIV-positive (SA group), 266 (26.7%) reported suicidal ideation only (without any active self-harm) after they were identified as HIV-positive (SI group), and 697 denied any SI or SA during this period (NSB group). As shown in Figure 1, 185 (18.6%) and 134 (13.5%) PLWH reported probable depression and probable anxiety, respectively.

The socio-demographic characteristics of the total sample and by group (i.e., SA, SI, and NSB) are presented in Table 1. Only 81 females were included in our study, but no significant gender difference was found among the three groups and their data remained in the analysis for this study. More than half of the recruited PLWH were middle-aged (with a median of 44 years old), living in rural areas, with a middle school or lower educational level, and an annual income per capita of <40,000 RMB. Nearly 35% of recruited PLWH were single, living alone, and employed as a farmer. There was a statistically significant age difference among the three groups. The PLWH in the SA group were older than those in the SI or NSB groups. There were no statistically significant differences in demographic variables among these three groups (see Table 1).

**Table 1:** The socio-demographic of total samples, Suicidal ideation only (SI), suicide attempt (SA), and without any suicidal behavior (NSB) groups of people living with HIV/AIDS (PLWH).

**Variables**	**Total (*****N*** **= 995)**		**SI (*****N*** **= 266)**		**SA (N = 32)**		**NSB (N = 697)**		**χ^2^/** ***H***	***p*** **-value**
	***N***	**%**	***N***	**%**	***N***	**%**	***N***	**%**		
**Gender**
Male	914	91.9	246	92.5	28	87.5	640	91.8	0.95	0.621
Female	81	8.1	20	7.5	4	12.5	57	8.2		
Age, median (IQR)	44	33–53	43	33–52	52	40–56	44	33–61	8.24	0.016^a^
**Age groups**
18–29	138	13.9	36	13.5	2	6.3	100	14.3	5.94	0.203
30–39	270	27.1	79	29.7	5	15.6	186	26.7		
≥40	587	59.0	151	56.8	25	78.1	411	59.0		
**Marital status**
Never married	345	34.7	102	38.3	10	31.3	233	33.4	3.44	0.779
Married	426	42.8	108	40.6	15	46.9	303	43.5		
Divorced/widowed	221	22.2	55	20.7	7	21.9	159	22.8		
Unknown	3	0.3	1	0.4	0	0.0	2	0.3		
**Residence**
Rural	561	56.4	155	58.3	18	56.3	388	55.7	0.53	0.767
Urban	434	43.6	111	41.7	14	43.8	309	44.3		
**Ethnic**
Han	970	97.5	259	97.4	31	96.9	687	98.6	2.60	0.234
Others	25	2.5	7	2.6	1	3.1	10	1.4		
**Religious beliefs**
None	846	85.0	233	87.6	25	78.1	588	84.4	3.37	0.465
Buddhism	106	10.7	23	8.6	5	15.6	78	11.2		
Other religious beliefs	43	4.3	10	3.8	2	6.3	31	4.4		
**Education level**
Middle/Technical secondary school or lower	573	57.6	141	53.0	18	56.3	414	59.4	4.12	0.390
High school/Vocational high school	326	32.8	93	35.0	11	34.4	222	31.9		
College/University or higher	96	9.6	32	12.0	3	9.4	61	8.8		
**Work status**
Farmer	352	35.4	111	41.7	8	25	233	33.4	9.30	0.054
Unemployed	56	5.6	17	6.4	3	9.4	36	5.2		
Others	587	59.0	138	51.9	21	65.6	428	61.4		
**Annual income per capita**
<40,000 RMB	564	56.7	143	53.8	21	65.6	400	57.4	2.11	0.348
≥40,000 RMB	431	43.3	123	46.2	11	34.4	297	42.6		
**Living situation**
Alone	343	34.5	98	36.8	12	37.5	233	33.4	1.13	0.569
Living with others	652	65.5	168	63.2	20	62.5	464	66.6		

*χ^2^: Chi square value; H: Kruskal–Wallis value*.

a *Tukey-type test was used to conduct multiple comparison, SA > NSB (q = 2.70, p < 0.05), SA > SI (q = 2.97, p < 0.01)*.

### Comparison of HIV Related Clinical Features and Psychosocial Factors

The HIV-related clinical features, included in the data from the CDC system, were missing from 2 out of 995 PLWH. As a result, we used the remaining HIV-related data from the 993 PLWHs for these data analyses. The differences of these clinical features did not reach statistical significance, except for CD4 counts (see Table 2). Overall, the HIV transmission route was strongly associated with a history of same-sex sexual behavior (Phi coefficient = 0.99, *p* < 0.001).

**Table 2:** HIV-related clinical features of total samples, SI, SA, and NSB groups of PLWH^*a*^.

**Variables**	**Total** ***(N*****=993)**		**SI (*****N*** **= 265)**		**SA (*****N*** **= 32)**		**NSB (*****N*** **= 696)**		**χ^2^/** ***H***	***p*** **-value**
	***N***	**%**	***N***	**%**	***N***	**%**	**N**	**%**		
**Same-sex sexual behavior**
Yes	567	57.0	165	62.3	16	50.0	386	55.5	4.31	0.116
No	426	42.8	100	37.7	16	50.0	310	44.5		
**Venereal disease history**
Yes	131	13.2	30	11.3	8	25.0	93	13.4	4.93	0.262
No	827	83.3	224	84.5	24	75.0	579	83.2		
Unknown	35	3.5	11	4.2	0	0.0	24	3.4		
**HIV transmission**
Same-sex sexual behavior	561	56.5	165	62.3	15	46.9	381	54.7	5.66	0.059
Others	432	43.5	100	37.7	17	53.1	315	45.3		
**CD4 test**
Yes	980	98.5	261	98.5	32	100.0	687	98.7	0.14	0.844
No	13	1.3	4	1.5	0	0.0	9	1.3		
**CD4 counts (cells/mm** ^**3**^ **), median (IQR)** ^***b***^	450	274–630	431	258–629.5	332	230.5–607.3	467	290–835.6	6.93	0.031
<200	145	14.6	50	19.2	5	15.6	90	13.1	6.95	0.139
200–500	417	41.9	109	41.8	16	50.0	292	42.5		
>500	418	42.0	102	39.1	11	34.4	305	44.4		
**With AIDS diagnosis**
Yes	418	42.1	120	45.3	16	50.0	282	40.5	2.64	0.268
No	575	57.9	145	54.7	16	50.0	414	59.5		

*χ^2^: Chi square value; H: Kruskal–Wallis value*.

a *Two out of 995 included PLWH missed data of HIV-related clinical features*.

b *The total sample of taking CD4 test was 980*.

In terms of depression and anxiety, the proportions of probable depression (i.e., PHQ-9 scored 10 or above) and probable anxiety (GAD-7 scored 10 or above) in the SA and SI groups were significantly higher than those in the NSB. The SA group reported higher HIV-related stress or stigma than the NSB group, and the SI group reported similar tendencies as the SA group. Lower social support and quality of life were found in the SA and SI groups compared with those in the NSB group. Differences of the above six psychosocial variables between SA and SI groups were not statistically significant (refer to Table 3).

**Table 3:** Psychosocial characteristics of total samples, SI, SA, and NSB groups of PLWH.

**Variables**	**Total (*****N*** **= 995)**		**SI (*****N*** **= 266)**		**SA (*****N =*** **32)**		**NSB (*****N*** **= 697)**		***χ^2^***	***p*** **-value**	**Multiple comparisons^a^**
	***N***	**%**	***N***	**%**	***N***	**%**	***N***	**%**			
**PHQ-9 scores (0–27)**											
No depression (<10)	810	81.4	141	53.0	16	50.0	653	93.7	232.00	<0.001	SA > C (*q* = 8.36, *p* <0.001)
Probable depression (≥10)	185	18.6	125	47.0	16	50.0	44	6.3			SI > C (*q* = 19.64, *p* <0.001)
**GAD-7 scores (0–21)**											
No anxiety (<10)	861	86.5	171	64.3	18	56.3	672	96.4	196.50	<0.001	SA > C (*q* = 8.39, *p* <0.001)
Probable anxiety (≥10)	134	13.5	95	35.7	14	43.8	25	3.6			SI > C (*q* = 17.63, *p* <0.001)
**Variables**	**Median**	***IQR***	**Median**	***IQR***	**Median**	***IQR***	**Median**	***IQR***	***H***	***p*** **-value**	**Multiple comparisons ^b^**
HIV-related stress	13	6–20	23	14–34	26.5	11.3–35.5	10	4–16	215.00	<0.001	SA > C (*q* = 5.74, *p* <0.001)
											SI > C (*q* = 14.06, *p* <0.001)
HIV-related stigma	11	8–13	13	11–14	13	10.3–14	10	7–14	114.80	<0.001	SA > C (*q* = 4.37, *p* <0.001)
											SI > C (*q* = 10.20, *p* <0.001)
Social support	25	20–31	22	17–28	20	18–26	26.5	21–33	59.00	<0.001	SA < C (*q* = 3.81, *p* <0.001)
											SI < C (*q* = 7.04, *p* <0.001)
Life quality	15	12–18	13	10–15	13	10–16.8	16	13–18	113.80	<0.001	SA < C (*q* = 3.64, *p* <0.001)
											SI < C (*q* = 10.37, *p* <0.001)

*χ^2^: Chi square value; H: Kruskal–Wallis value*.

a *Using a non-parametric method*.

b *Using Tukey-type method*.

### Multivariate Analysis of Factors Associated With SI and SA

The AORs for the three-way comparison (SI and SA groups compared with NSB group and with each other) of psychosocial variables (Model 1) and HIV-related clinical features (Model 2) are presented in Table 4. Both models were adjusted for demographic variables (age, gender, residence, annual income, and educational status) using multinomial logistic regression. Model 1 revealed that after adjusting for demographic variables: (a) depression, anxiety, and HIV-related stigma were associated with SI and SA; (b) HIV-related stress and quality of life were associated with SI but not with SA; and (c) although the associations of SA and probable depression or probable anxiety (AORs 4.20 and 5.68) were almost two times the associations of SI and probable depression or anxiety (AORs 2.56 and 2.63), these differences did not reach statistical significance. In addition, the SA group was indistinguishable from the SI group across the other psychosocial factors (see Table 4).

**Table 4:** Adjusted odds ratios (AORs) and 95% Confidence Intervals (95% CIs) of factors associated with SI or SA among PLWH using multinomial logistic regression^a^.

	**AOR (95% CI)**					
	**SI v. NSB (reference)**		**SA V. NSB (reference)**		**SA v. SI (reference)**	
**Variables**	**Model 1^b^**	**Model 2^c^**	**Model 1^b^**	**Model 2^c^**	**Model 1^b^**	**Model 2^c^**
Probable depression (≥10)	**2.56** (1.62–4.04)	**2.43** (1.51–3.88)	**4.20** (1.16–15.2)	**4.44** (1.22–16.1)	1.64 (0.44–6.14)	1.83 (0.49–6.88)
Probable anxiety (≥10)	**2.63** (1.69–4.09)	**2.80** (1.77–4.42)	**5.68** (1.59–20.3)	**5.62** (1.55–20.4)	2.16 (0.59–7.97)	2.01 (0.54–7.52)
High HIV-related stress (≥14)	**1.92** (1.29–2.85)	**1.91** (1.27–2.88)	1.24 (0.49–3.14)	1.17 (0.45–3.04)	0.65 (0.25–1.66)	0.62 (0.23–1.62)
High HIV-related stigma (≥12)	**1.99** (1.39–2.86)	**2.05** (1.41–2.98)	**2.59** (1.09–6.14)	**2.65** (1.09–6.39)	1.30 (0.54–3.11)	1.29 (0.53–3.15)
High social support (≥26)	0.80 (0.56–1.14)	0.83 (0.57–1.20)	0.71 (0.31–1.60)	0.68 (0.30–1.56)	0.89 (0.39–2.01)	0.82 (0.36–1.88)
High life quality (≥16)	**0.59** (0.40–0.87)	**0.56** (0.37–0.84)	0.94 (0.38–2.31)	0.93 (0.37–2.36)	1.59 (0.64–3.98)	1.68 (0.65–4.33)
HIV transmission
Same-sex sexual behavior		0.70 (0.47–1.02)		1.14 (0.50–2.62)		1.64 (0.72–3.76)
Others		1		1		1
Venereal disease (VD) history		0.97 (0.59–1.61)		2.19 (0.86–5.54)		2.25 (0.90–5.65)
With AIDS diagnosis		1.10 (0.72–1.70)		1.19 (0.48–2.94)		1.08 (0.44–2.66)
CD4 counts (cells/mm^3^)
<200		0.82 (0.44–1.53)		0.90 (0.23–3.50)		1.10 (0.29–4.23)
200–500		0.93 (0.62–1.39)		0.72 (0.30–1.75)		0.78 (0.32–1.88)
>500		1		1		1

*SA, Participants with suicide attempt after identified as HIV+; SI, Participants with suicidal ideation only after identified as HIV+; NSB, Participants with neither suicide attempt nor suicidal ideation after identified as HIV+*.

a *Data in bold indicated statistical significance*.

b *Model 1 included psychosocial variables (i.e., depression, anxiety, HIV-related stress, stigma, and life quality), after adjusted for age, gender, residence, annual income, and education*.

c *Model 2 included psychosocial variables (i.e., depression, anxiety, HIV-related stress, stigma and life quality) and HIV-related clinical variables (i.e., HIV-transmission, AIDs diagnosis, VD history, and CD4 counts), after adjusted for age, gender, residence, annual income, and education. We excluded the variable of same-sex sexual behaviors because it was significantly correlated with the HIV transmission via same-sex sexual behaviors*.

When HIV-related clinical features were included (Model 2), the AORs of associations of psychosocial factors and SI or SA were similar to that in Model 1, and the three groups of PLWH did not differ in HIV-related clinical features (see Table 4).

## Discussion

In this study, we recruited 78.6% (995/1,266) of the PLWH who lived and were registered in a middle-income municipality in central west China. Results indicate that 18.6% (185 cases) and 13.5% (134 cases) of the included PLWH reported probable depression (PHQ-9 ≥ 10) or probable anxiety (GAD-7 ≥ 10), respectively. In addition, 26.7% (266 cases) and the other 3.2% (32 cases) reported SI (without SA) or made SAs after they were diagnosed as HIV-positive. After adjusting for demographic variables, both SI and SA were associated with probable depression, probable anxiety, and HIV-related stigma. Similarly, the SI was associated with HIV-related stress and quality of life. The HIV-related clinical features were not associated with either SI or SA. Of note, the SA group did not differ from the SI group on any of the psychosocial or HIV-related clinical features.

Our findings confirm the mental health needs of PLWH, which have been documented in previous studies (5, 6, 13, 25–29). The prevalence of such mental health problems, however, have significantly varied across different studies, e.g., ranging from 18.3 to 86.9% of PLWH suffered from depression (25), 11.1–97.5% suffered from anxiety (5), and 3.3–32.6% and 3.75–26% reported SI or SA, respectively (6, 13, 26–29). This variation might be partly attributed to the use of different screening tools, cut-off points for depression and anxiety, time intervals for the occurrence of SI and SA, and the settings from which PLWH populations are recruited. For example, a broad range of instruments was used for mental health screening, e.g., 9 and 8 different questionnaires for depression and anxiety, respectively (5). In one study, a cut-off of 5 on the PHQ-9 was used to screen depression in a previous study (25). In other studies, the lifetime prevalence of SI or SA was higher in those studies which assessed over a shorter time interval (e.g., identification of HIV+ in last 6 months versus in last two weeks) (5, 6, 27, 28). Inpatient PLWH reported a relatively lower prevalence of SI (26) than those who are outpatients. Therefore, our relatively low prevalence of depression and anxiety among PLWH may be a result of the rigid criteria of our study. We assessed depression and anxiety in the past 2 weeks, and a criterion equal to or higher than 10 was used to identify probable depression or anxiety, which is a more conservative estimate compared to some prior studies (17, 18). In terms of SI or SA, only those that occurred after HIV+ diagnosis were considered in our study, as we were interested in the time period after receiving this diagnosis.

In terms of correlates of SI and SA in PLWH, the main results in our study are similar to previous studies reporting that individuals with high levels of stress and stigma and low social support are more likely to engage in SI (9, 14, 30, 31) and SA (14, 31). In our study, the PLWH with SI and SA were more likely to endorse probable depression, probable anxiety, and higher HIV-related stigma than those with NSB. The PLWH with SI was more likely to experience high HIV-related stress and lower quality of life than PLWH with NSB. The findings suggest that mental health problems and HIV-related stress and stigma are key targets for health promotion and suicide prevention in PLWH.

Previous studies have documented correlations of suicidal behaviors and HIV-related clinical features, including low CD4 cell counts (26), suspension of ART (26), and the presence of an AIDS diagnosis (29). In our study, no associations of HIV-related clinical features with SI or SA were statistically significant. This may be due to our sample characteristics. Similar to other studies that also did not find any associations (14, 30, 32–34), the recruited PLWH in our study were community residents (registered as HIV+ in CDC HIV system), largely male-identifying, and receiving ART. To clarify such associations further, future prospective studies might recruit specific cohorts of PLWH.

Many risk factors (living in rural areas and low income in China) and protective factors (religious beliefs, social support, and high life quality) of suicidal behavior have been identified for the general population (23, 35). Nevertheless, our study suggests that the same risk and protective factor profile cannot be generalized to PLWH, with one exception: high quality of life and SI (AOR = 0.56). Experiences unique to PLWH, i.e., social isolation, discrimination, and daily life inconveniences due to the HIV infection, may contribute to such differences. Social support, for example, does not have the same buffering effect on depressive and anxiety symptoms among PLWH as seen in the general population, and this may be HIV-specific, such as stress-related to confidentiality (36). Also, low quality of life did not appear to be a distinct risk factor for SA among PLWH. These results suggest that suicide prevention strategies for PLWH should focus on reducing stigma and other HIV+ specific concerns, along with depression and anxiety.

The present study, to the best of our knowledge, is the first to compare correlation between SA and SI among PLWH in China. Results did not approach statistical significance differences for the psychosocial or HIV-related clinical features between PLWH with SA and those with SI only. In the Turecki and Brent model for suicide risk (35), depression is one of the precipitating factors for SI and anxiety, while other factors moderate the pathway from SI to suicidal acts in the general population. Within special populations with a high prevalence of depression or anxiety, however— including prisoners (37), depressed patients (38), and PLWH in our study—depression and anxiety are not as helpful for distinguishing between those who report SA and those who report SI only. The findings suggest that more sensitive tools for screening depression and anxiety are necessary to estimate the potential risk of SA in special populations. The HIV-related stress and stigma and quality of life did not distinguish between SA groups and SI groups in PLWH as they do and have done in our current and previous studies (9, 29, 30). This indicates that HIV-related features and quality of life might contribute to the emergence of SI but may not play key roles in the transition from SI to SA among PLWH. If such factors are identified, they might be the key targets for suicide prevention in PLWH. Other factors should be taken into consideration in future studies, such as the role of social media use (39) and exposure to others' suicidal behaviors among prisoners (37), among others.

There are several limitations to this study. First, our study is a cross-sectional design which limits our ability to make causal inferences between SI or SA and other psychosocial variables. Second, the sample size is limited, as only 32 participants reported SA after an HIV-positive diagnosis, resulting in an increased chance for type II errors. Third, only PLWH who resided in and registered in the Baoji municipality were included in our study and as such, our results may not be as generalizable to other regions. Fourth, <10% of the recruited participants identified as female, and our findings may not be generalizable to female PLWH. Fifth, factors not included in our study might be associated with SI or SA, such as a course of HIV+, ART treatment, HIV-related complications, side effects of HIV-related treatment, and HIV-associated neurocognitive disorder (HANDS). Sixth, HIV-positive course of illness varied across different participants, and those with longer illness courses may have a higher probability of experiencing SI or SA. Although there is some conflicting evidence, as previous studies reported that PLWH with longer courses coped with stress better than those with shorter courses (40).

Our findings suggest several considerations for tailoring suicide prevention strategies for PLWH in China. First, depression and anxiety are persistent mental health problems and are associated with SI or SA in PLWH. Given the low utilization of mental health services in PLWH in China (40), mental health promotion programs in PLWH should include depression and anxiety treatment elements specific to reducing suicidal ideation. Second, tailored suicide prevention strategies are necessary for PLWH, given that high social support may not serve as a protective factor for SI or SA in this population. Health education and other programs to reduce HIV-related stress and stigma should be included as key elements. Third, more sensitive tools for screening depression and anxiety, and clarification of potential risk factors for SAs are still needed. In conclusion, this study contributes to our understanding of mental health problems in PLWH in Central West China. It will be important to deliver effective and low-cost psychological services and suicide prevention strategies that are specific to PLWH.

## Data Availability Statement

The original contributions presented in the study are included in the article/supplementary material, further inquiries can be directed to the corresponding author/s.

## Ethics Statement

The studies involving human participants were reviewed and approved by the Institutional Review Board of the Beijing HuiLongGuan Hospital. The patients/participants provided their written informed consent to participate in this study. Written informed consent was obtained from the individual(s) for the publication of any potentially identifiable images or data included in this article.

## Author Contributions

XW, CY, YT, and WZ designed the study. CY, YT, JG, WZ, ZL, HL, and FD administered the investigation. XW and WZ wrote the initial draft of the article. JW assisted in the preparation of the article. YT, WZ, YY, and YW have contributed to the collection and interpretation of data. YT, YY, and NL have reviewed the analyses, data interpretation, and edited the final drafts of the manuscript. All authors approved the article and agreed to be accountable for all aspects of the work in ensuring that questions related to the accuracy or integrity of any part of the work are appropriately investigated and resolved.

## Funding

This work was supported by the National Natural Science Foundation of China [82071546] and the Beijing Hospitals Authority Clinical Medicine Development of Special Funding Support [ZYLX202130].

## Conflict of Interest

The authors declare that the research was conducted in the absence of any commercial or financial relationships that could be construed as a potential conflict of interest.

## Publisher's Note

All claims expressed in this article are solely those of the authors and do not necessarily represent those of their affiliated organizations, or those of the publisher, the editors and the reviewers. Any product that may be evaluated in this article, or claim that may be made by its manufacturer, is not guaranteed or endorsed by the publisher.

## References

[1] NCAIDS NCSTD CDC C. Update on the AIDS/STD epidemic in China in the third quarter of 2018. Chin J AIDS STD. (2018) 24:1075. 10.13419/j.cnki.aids.2018.11.01

[2] May M Gompels M Delpech V Porter K Post F Johnson M. Impact of late diagnosis and treatment on life expectancy in people with HIV-1: UK Collaborative HIV Cohort (UK CHIC) Study. BMJ. (2011) 343:d6016. 10.1136/bmj.d6016 21990260 PMC3191202

[3] Brandt R. The mental health of people living with HIV/AIDS in Africa: a systematic review. Afr J AIDS Res. (2009) 8:123–33. 10.2989/AJAR.2009.8.2.1.853 25875564

[4] Schadé A van Grootheest G Smit JH. HIV-infected mental health patients: characteristics and comparison with HIV-infected patients from the general population and non-infected mental health patients. BMC Psychiatry. (2013) 13:35. 10.1186/1471-244X-13-35 23343356 PMC3577506

[5] Niu L Luo D Liu Y Silenzio VM Xiao S. The mental health of people living with HIV in China, 1998–2014: a systematic review. PLoS ONE. (2016)11:e0153489. 10.1371/journal.pone.0153489 27082749 PMC4833336

[6] Pelton M Ciarletta M Wisnousky H Lazzara N Manglani M Ba DM. Rates and risk factors for suicidal ideation, suicide attempts and suicide deaths in persons with HIV: a systematic review and meta-analysis. Gen Psychiatry. (2021) 34:e100247. 10.1136/gpsych-2020-100247 33912798 PMC8042999

[7] Chen L Pan X Ma Q Yang J Xu Y Zheng J. HIV cause-specific deaths, mortality, risk factors, and the combined influence of HAART and late diagnosis in Zhejiang, China, 2006–2013. Sci Rep. (2017) 7:42366. 10.1038/srep42366 28198390 PMC5309804

[8] Chen F Cai C Wang S Qin Q Jin Y Li D. Trends in suicide mortality among people with HIV after diagnosis during 2012–18: a retrospective, national cohort study in China. Lancet HIV. (2022) 9:e102–11. 10.1016/S2352-3018(21)00316-7 35120631

[9] Liu Y Niu L Wang M Chen X Xiao S Luo D. Suicidal behaviors among newly diagnosed people living with HIV in Changsha, China. AIDS Care. (2017) 29:1359–63. 10.1080/09540121.2017.1338653 28593797

[10] Wang YY Dong M Zhang Q Xu D Zhao J Ng CH. Suicidality and clinical correlates in Chinese men who have sex with men (MSM) with HIV infection. Psychol Health Med. (2019) 24:137–43. 10.1080/13548506.2018.1515495 30175922

[11] Brown LA Majeed I Mu W McCann J Durborow S Chen S. Suicide risk among persons living with HIV. AIDS Care. (2021) 33:616–22. 10.1080/09540121.2020.1801982 32741212 PMC7855619

[12] Bi F Luo D Huang Y Chen X Zhang D Xiao S. The relationship between social support and suicidal ideation among newly diagnosed people living with HIV: the mediating role of HIV-related stress. Psychol Health Med. (2021) 26:724–34. 10.1080/13548506.2020.1761987 32400173

[13] Wang W Chen X Yan H Yu B Li S. Association between social capital and suicide ideation, plan and attempt among men living with HIV China. J Affect Disord. (2021) 280(Pt A):173–9. 10.1016/j.jad.2020.11.088 33212409

[14] Lu H Sheng W Liao S Chang N Wu P Yang Y Hsiao F. The changes and the predictors of suicide ideation and suicide attempt among HIV-positive patients at 6–12 months post diagnosis: a longitudinal study. J Adv Nurs. (2019) 75:573–84. 10.1111/jan.13883 30334591

[15] Jia C Mehlum L Qin P. AIDS/HIV infection, comorbid psychiatric illness, and risk for subsequent suicide: a nationwide register linkage study. J Clin Psychiatry. (2012) 73:1315–21. 10.4088/JCP.12m07814 23059105

[16] De Leo D Goodfellow B Silverman M Berman A Mann J Arensman E. International study of definitions of English-language terms for suicidal behaviours: a survey exploring preferred terminology. BMJ Open. (2021) 11:e043409. 10.1136/bmjopen-2020-043409 33563622 PMC7875264

[17] Bian C He X Qian J Wu W Li C. The reliability and validity of a modified patient health questionnaire for screening depressive syndrome in general hospital outpatients. J Tongji Univ. (2009) 30:136–40.

[18] He X Li C Qian J Cui H Wu W. Reliability and validity of a generalized anxiety disorder scale in general hospital outpatients. Shanghai Arch Psychiatry. (2010) 22:200–203.

[19] Niu L Qiu Y Luo D Chen X Wang M Pakenham KI. Cross-culture validation of the HIV/AIDS stress scale: the development of a revised Chinese version. PLoS ONE. (2016). 11:e0152990. 10.1371/journal.pone.0152990 27043134 PMC4820124

[20] Berger BE Ferrans CE Lashley FR. Measuring stigma in people with HIV: psychometric assessment of the HIV stigma scale. Res Nurs Health. (2001) 24:518–29. 10.1002/nur.10011 11746080

[21] Li L Guo Y. Validity and reliability of the simplified Berger HIV Stigma Scale in multiethnic areas. Chin Mental Health J. (2010). 24:854–8. 10.3969/j.issn.1000-6729.2010.11.014

[22] Xiao S. The social support scale. Chin Mental Health J. (1999) 13(suppl):127–33.

[23] Liu Y Tong Y Yin Y Li L Wu M. Relations of suicide and suicide attempts to social support and quality of life in rural China. Chin Mental Health J. (2020) 34:408–15. 10.3969/j.issn.1000-6729.2020.5.005

[24] Zar HG. Biostatistical Analysis. 4th ed. Upper Saddle River, NJ: Prentice Hall (1999).

[25] Wang T Fu H Kaminga AC Li Z Guo G Chen L. Prevalence of depression or depressive symptoms among people living with HIV/AIDS in China: a systematic review and meta-analysis. BMC Psychiatry. (2018) 18:160. 10.1186/s12888-018-1741-8 29855289 PMC5984474

[26] Durham MD Armon C Mahnken JD Novak RM Palella FJ Jr Tedaldi E. Rates of suicidal ideation among HIV-infected patients in care in the HIV Outpatient Study 2000–2017, USA. Prev Med. (2020) 134:106011. 10.1016/j.ypmed.2020.106011 32027915 PMC10132173

[27] López JD Shacham E Brown T. Suicidal ideation persists among individuals engaged in HIV care in the era of antiretroviral therapy. AIDS Behav. (2018) 22:800–5. 10.1007/s10461-016-1666-5 28063073

[28] Tsegay L Ayano G. The prevalence of suicidal ideation and attempt among young people with HIV/AIDS: a systematic review and meta-analysis. Psychiatr Q. (2020) 91:1291–304. 10.1007/s11126-020-09851-1 32960412

[29] Cooperman NA Simoni JM. Suicidal ideation and attempted suicide among women living with HIV/AIDS. J Behav Med. (2005) 28:149–56. 10.1007/s10865-005-3664-3 15957570

[30] Wang W Xiao C Yao X Yang Y Yan H Li S. Psychosocial health and suicidal ideation among people living with HIV/AIDS: a cross-sectional study in Nanjing, China. PLoS ONE. (2018) 13:e0192940. 10.1371/journal.pone.0192940 29470532 PMC5823403

[31] Bitew H Andargie G Tadesse A Belete A Fekadu W Mekonen T. Suicidal ideation, attempt, and determining factors among HIV/AIDS patients, Ethiopia. Depress Res Treat. (2016) 2016:8913160. 10.1155/2016/8913160 27747101 PMC5055996

[32] Chikezie UE Otakpor AN Kuteyi OB James BO. Suicidality among individuals with HIV/AIDS in Benin City, Nigeria: a case-control study. AIDS Care. (2012) 24:843–5. 10.1080/09540121.2011.645008 22272812

[33] Passos SM Souza LD Spessato BC. High prevalence of suicide risk in people living with HIV: who is at higher risk? AIDS Care. (2014) 26:1379–82. 10.1080/09540121.2014.913767 24797027

[34] Hentzien M Cabie A Pugliese P Billaud É Poizot-Martin I Duvivier C. Factors associated with deaths from suicide in a French nationwide HIV-infected cohort. HIV Med. (2018) 19:551–8. 10.1111/hiv.12633 29856132

[35] Turecki G Brent DA. Suicide and suicidal behavior. Lancet. (2016) 387:1227–39. 10.1016/S0140-6736(15)00234-2 26385066 PMC5319859

[36] Huang Y Luo D Chen X Zhang D Huang Z Xiao S. HIV-related stress experienced by newly diagnosed people living with HIV in China: a 1-year longitudinal study. Int J Environ Res Public Health. (2020) 17:268. 10.3390/ijerph17082681 32295107 PMC7216022

[37] Favril L O'Connor RC Hawton K Vander Laenen F. Factors associated with the transition from suicidal ideation to suicide attempt in prison. Eur Psychiatry. (2020) 63:e101. 10.1192/j.eurpsy.2020.101 33183374 PMC7737175

[38] Wagner G Li M Sacchet MD Richard-Devantoy S Turecki G Bär KJ. Functional network alterations differently associated with suicidal ideas and acts in depressed patients: an indirect support to the transition model. Transl Psychiatry. (2021) 11:100. 10.1038/s41398-021-01232-x 33542184 PMC7862288

[39] Liu X Huang J Yu NX Li Q Zhu T. Mediation effect of suicide-related social media use behaviors on the association between suicidal ideation and suicide attempt: cross-sectional questionnaire study. J Med Internet Res. (2020) 22: e14940. 10.2196/14940 32343249 PMC7218592

[40] Niu L Luo D Chen X Wang M Zhou W Zhang D. Longitudinal trajectories of emotional problems and unmet mental health needs among people newly diagnosed with HIV in China. J Int AIDS Soc. (2019) 22:e25332. 10.1002/jia2.25332 31424617 PMC6699581

